# A systematic review and meta-analysis of fusion rate enhancements and bone graft options for spine surgery

**DOI:** 10.1038/s41598-022-11551-8

**Published:** 2022-05-09

**Authors:** Wagner M. Tavares, Sabrina Araujo de França, Wellingson S. Paiva, Manoel J. Teixeira

**Affiliations:** 1 Department of Research of IPSPAC-Instituto Paulista de Saúde para Alta Complexidade, 215 Alameda Terracota, cj 407, Sao Caetano do Sul, SP 09531-190 Brazil; 2 Institute of Neurology, University of São Paulo, 255 Dr. Enéas de Carvalho Aguiar avenue, Cerqueira César, São Paulo, SP 05403-900 Brazil

**Keywords:** Neuroscience, Medical research, Neurology

## Abstract

Our study aimed to evaluate differences in outcomes of patients submitted to spinal fusion using different grafts measuring the effectiveness of spinal fusion rates, pseudarthrosis rates, and adverse events. Applying the Preferred Reporting Items for Systematic Reviews and Meta-Analyses statement, this systematic review and meta-analysis identified 64 eligible articles. The main inclusion criteria were adult patients that were submitted to spinal fusion, autologous iliac crest (AIC), allograft (ALG), alloplastic (ALP; hydroxyapatite, rhBMP-2, rhBMP-7, or the association between them), and local bone (LB), whether in addition to metallic implants or not, was applied. We made a comparison among those groups to evaluate the presence of differences in outcomes, such as fusion rate, hospital stay, follow-up extension (6, 12, 24, and 48 months), pseudarthrosis rate, and adverse events. Sixty-four studies were identified. LB presented significantly higher proportions of fusion rates (95.3% CI 89.7–98.7) compared to the AIC (88.6% CI 84.8–91.9), ALG (87.8% CI 80.8–93.4), and ALP (85.8% CI 75.7–93.5) study groups. Pseudarthrosis presented at a significantly lower pooled proportion of ALG studies (4.8% CI 0.1–15.7) compared to AIC (8.6% CI 4.2–14.2), ALP (7.1% CI 0.9–18.2), and LB (10.3% CI 1.8–24.5). ALP and AIC studies described significantly more cases of adverse events (80 events/404 patients and 860 events/2001 patients, respectively) compared to LB (20 events/311 patients) and ALG (73 events/459 patients). Most studies presented high risk-of-bias scores. Based on fusion rates and adverse events proportions, LB showed a superior trend among the graft cases we analyzed. However, our review revealed highly heterogeneous data and a need for more rigorous studies to better address and assist surgeons’ choices of the best spinal grafts.

## Introduction

Spine fusions have become a frequent treatment choice for distinct spinal pathologies over the past four decades. Rajaee et al.^1^ estimated a spinal fusion rate increase from 64.5 cases per 100,000 adults in 1998 to 135.5 cases per 100,000 in 2008. Primary cervical and lumbar fusion increased from 73.717 and 77.682 in 1998 to 157.966 and 210.4047 in 2008, respectively^1^. In the United States, lumbar degenerative disc disease surgical interventions increased from 21,223 in 2000 to 55,467 in 2009^2^. Similarly, the market for spinal implants and devices was estimated at $7 billion in sales between 2013 and 2014^3^, reflecting an increase in material availability. However, the current literature insufficiently confirms the superiority of one intervention or graft^4,5^.

Like any other surgical intervention, spine fusions can lead to unexpected outcomes, such as pseudarthrosis or other adverse events. *Pseudarthrosis* can be defined as a solid fusion failure, whether symptomatic or asymptomatic, that can increase the risk of neurologic symptoms, material failure, and deformity^6,7^. To make appropriate decisions, surgeons must weigh the effectiveness versus costs of each graft type.

Autologous iliac crest (AIC) graft has been considered the gold standard treatment for spinal fusion because of its histocompatible and non-immunogenic properties, presenting higher amounts of cancellous bone, growth factors, and pluripotent cells related to osteoinduction, osteogenesis, and osteoconduction^8–10^. Unfortunately, spinal fusions with AIC have been associated with several morbidities, such as a higher incidence of infection, donor site pain, hematoma development, increased operative time, and blood loss^11–16^.

As consequence of AIC drawbacks, alternative grafts have been developed, and these alternatives are increasingly diverse and available. Such materials can be classified as *extender*, *enhancer*, or *substitute* grafts^17,18^. An *extender* decreases the need for large amounts of autologous bone grafting (ABG) while offering the same bone formation properties as AIC^17,18^. An *enhancer* is a material combined with ABG to increase successful fusion rates compared to ABG alone^17,18^. A *substitute* replaces an ABG and presents the same or higher healing success rates compared to ABG alone^17,18^.

These materials are often assembled in various proportions to achieve spinal fusion^17^. However, allograft (ALG) and alloplastic (ALP) grafts are foreign bodies that carry some inherent risks. Considering their pros and cons, AIC use is favorable since AIC need not be associated with other grafts to achieve reliable results^19^. One frequently used alternative is local bone (LB), however, to our best knowledge, previous studies have only compared autologous bone graft with ALP and ALG. They failed to make a subdivision of autologous bone graft in LB and AIC. This is crucial since if it is possible to avoid using AIC, or other nonlocal bone, the post operatory morbidity, especially residual pain and an extra wound/scar, can be avoided.

## Methods

This study was conducted according to the Preferred Reporting Items for Systematic Reviews and Meta-Analyses (PRISMA) statement^20^. A comprehensive web-based literature search was conducted through to January 2021, using three databases (Lilacs, PubMed, and Cochrane), by two independent authors (SAF and ASMS), without publication-language restrictions. For all databases, controlled vocabulary and text word searches were performed, using a combination of the keywords: “spinal fusion AND autograft AND spinous process”, “spinal fusion AND autograft AND spinal lamina”, “spinal fusion AND autograft AND iliac crest”, “spinal fusion AND heterograft”, “spinal fusion AND allograft AND spinous process”, “spinal fusion AND allograft AND spinal lamina”, “spinal fusion AND allograft AND iliac crest”. Our search was direct toward adult patients that were submitted to spinal fusion, which ALG, ALP, LB or AIC was applied. Due to an increase in medical devices availability nowadays, we compared these groups between them to evaluate the presence of differences or superiority in outcomes, such as fusion rate, hospital stay, follow-up extension (6, 12, 24, and 48 months), pseudarthrosis rate, and adverse events.

Titles, abstracts, and full-text studies were reviewed according to pre-established criteria, and then the relevant data were extracted. Discrepancies were resolved by consensus with the remainder of the research team. This study’s inclusion and exclusion criteria are presented in Table 1. Retrospective studies, prospective analyses, randomized clinical trials, and case series were included in this review. The cut-off date for the review was January 31, 2021.

**Table 1:** Inclusion and exclusion criteria.

Inclusion	Exclusion
Full-texts available	Studies which applied more than one graft per procedure, with/without adjuvants (besides simultaneous using of cage, plate and/or screws)
Articles in English, Portuguese or French	
Adult human subjects (≥ 18 years)	
Studies with more than five subjects	Papers that did not report fusion rate
Radiologic confirmation of fusion per patient	Animal, cadaver, and biomechanical studies
One graft type or with an exclusively association with one metallic implant (cage, plate and/or screws)	Case reports, commentaries, editorials and reviews

### Data extraction

The following data were abstracted from all included studies: *study design*, *year*, *patient demographics*, *preoperative assessment*, *intraoperative information*, *postoperative assessment*, *hospital stay*, *follow-up extension*, *fusion rate*, *pseudarthrosis rate* (comprising reported data for nonunion and pseudarthrosis), and *adverse events* (graft-related, infections, and neurological). Data were partially (one graft-type group of interest) or fully (all graft-type groups in the study) extracted from comparative studies, in accordance with our inclusion criteria. Two investigators (SAF and ASMS) independently performed a systematic review of all identified citations. No attempts were made to contact the authors of the reviewed studies to obtain missing or unreported data. Our main outcome of interest was fusion rates, and secondary outcomes included pseudarthrosis and adverse event rates.

### Risk of bias assessments and evaluations of validity

The quality of eligible studies and their risk of bias (RoB) were examined by two reviewers (SAF and ASMS) using the methodological index for non‐randomized studies (MINORS)^21^, and the Cochrane’s collaboration tool for assessing RoB^22^ in randomized controlled trials. The high risk of bias for RoB score for non-randomized studies was determined to be ≤ 8 (controlled group not present) or ≤ 12 (controlled group present). For randomized controlled trials, each domain was classified as *unclear bias*, *low RoB*, or *high RoB*.

### Heterogeneity assessments

Heterogeneity between studies was examined using the I^2^ statistic and the *P*-value for heterogeneity^23^. *Substantial heterogeneity* is defined as ≥ 50%^24^.

### Data analysis

Meta-analysis of proportions, using MedCalc 16.2.0, was performed to estimate an overall weighted proportion and its 95% confidence interval (CI) for each outcome of interest. MedCalc uses a Freeman–Tukey transformation to calculate summary proportions, weighted according to the number of patients described in each study. We determined the pooled proportion using a random-effects model. Data were summarized in tables and further stratified based on bone graft types (AIC, ALG, ALP, [comprising hydroxyapatite, rhBMP-2, rhBMP-7, titanium cages], and LB). The Kruskal–Wallis test was used to compare variables among the four groups, and post hoc analyses using Mann–Whitney *U* tests were performed to compare two groups. When multiple follow-up periods were available for a study, data from the last assessment were used for the combined analyses. Subsequently, the fusion rates stratified by bone graft substitutes (bone graft alone or combined with metallic implants), and follow-up periods (6, 12, 24, and 48 months) were further analyzed (subgroup analysis). Studies that did not report the timing of fusion rates assessments were excluded from this subgroup analysis. Further analysis (meta-regression) to identify factors related to fusion rates (surgical approach, pseudarthrosis, and adverse events) were unsuccessful because the methods used to report the data were inconsistent across studies.

## Results

### Study demographics

As designated by the PRISMA guidelines^20^, Supplementary Fig. 1 is a flowchart describing our database research, which identified 1535 studies. After passing a screening phase, 184 studies were fully reviewed, leading to 120 exclusions that are detailed in Supplementary Table 1. A total of 64 studies (4177 subjects) were selected for this systematic review. Analyses were performed by reorganizing studies according to graft material (with or without metallic implants) samples, resulting in 91 analyses (51 analyses regarding AIC, 9 analyses regarding ALG, 20 analyses regarding ALP, and 10 analyses regarding LB). PRISMA checklist can be consulted at Supplementary Table 2. According to MINORS^21^ and Cochrane’s collaboration tool for assessing RoB^22^, the majority of studies presented high RoB scores, as demonstrated on Supplementary Table 3 and Supplementary Figs. 2 and 3.

### Participant demographics

Patients’ and procedures’ characteristics are summarized in Table 2. Overall, patients’ main diagnoses for surgical intervention were degenerative diseases (78.8%). A thorough analysis of follow-up, procedure duration, blood loss, and hospital length of stays (LOS) was impaired due to a lack of systematic reports. Data were inconsistent across studies since none of the ALG articles specified hospital LOS. Similarly, some aspects had been exposed by a unique author, such as procedure time and blood loss in the ALG and ALP groups.

**Table 2:** Patient’s characteristics and clinical outcomes.

Aspects	AIC	ALG	ALP	LB
Number of patients	2529 (male: 1174; female: 1096; NI: 259)	516 (male: 231; female: 245; NI: 40)	766 (male: 311; female: 322; NI: 133)	366 (male: 185; female: 181)
Mean age, years	48.8 ± 10.5	46.6 ± 11.9	61.3 ± 9.6	52.6 ± 10.9
**Diagnosis**				
Degenerative disease	1927	442	593	333
Trauma	346	12	40	33
Neoplasm	6	4	2	NA
NI	247	40	101	NA
Others	3	18	30	NA
**Surgical region, number of patients, (%)**				
Occiptocervical and cervical	1232	470	375	238
Thoracolumbar and lumbar	1297	46	391	128
Follow-up, months	26.8 ± 8.6	21.1 ± 12.8	36.0 ± 6.5	38.7 ± 14.9
Duration of procedure, min	174.3 ± 54.0	243 ± 73.0^a^	138.0 ± 43.0^a^	154.4 ± 36.2
Blood loss, ml	509.1 ± 442.1	195 ± 89.0^a^	387.0 ± 100.0^a^	333.0 ± 141.0
Hospital stay, days	5.08 ± 2.9	NI	3.5 ± 2.4	7.8 ± 4.1

*AIC* Autologous iliac crest, *ALG* allograft, *ALP* alloplastic, *LB* local bone, *NA* not applied, *NI* not informed.

^a^Cited in one study.

### Pre- and post-operative assessments

Patient assessments were not reported systematically, making this study’s analysis difficult. Apart from distinct assessments during patients’ clinical courses, such as weight and height (in preoperative assessments) and Odom’s criteria (in postoperative assessments), pre- and post-operative assessments included matching analysis only for Japanese Orthopedic Association Score (JOA) and Nurick Grade reports in the AIC group. The same pattern was observed in the LB group (Frankel scale report) and ALP group (Frankel and JOA reports), as Table 3 shows.

**Table 3:** Patient’s pre and postoperative assessments.

Aspects	AIC	ALG	ALP	LB
**Pre-operative assessment**				
JOA	10.8 ± 1.8	NI	13.5 ± 1.7^a^	NI
Height	169.6 ± 12.3	NI	169.0 ± 3.3	NI
Weight	80.2 ± 20.9	NI	82.0 ± 5.3	NI
ODI Score	46.3 ± 12.2	NI	49.4 ± 10.7	30.2 ± 5.7
NDI	26.8 ± 1.1	NI	18.2 ± 11.6^a^	NI
Frankel, number of patients (total)	A:6; B:8; C:19; D:21; E:32; (86)	NI	A:1; B:2; C:10; D:24; E:15; (52)	A:1; B:0; C:3; D:8; E:21; (33)^a^
Pain VAS	7.2 ± 1.6	NI	7.6 ± 2.0	7.7 ± 1.4
**Post-operative assessment**				
JOA	15.3 ± 1.5	NI	16.4 ± 1.2^a^	NI
ODI Score	21.3 ± 7.9	NI	25.2 ± 2.6	13.6 ± 1.9
Frankel, number of patients (total)	A:2; B:3; C:2; D:13; E:45; (65)	NI	A:1; B:1; C:3; D:12; E:35; (52)	A:0; B:0; C:0; D:2; E:31; (33)^a^
Odom’s Criteria number of patients (total)	E + G: 269; F + P: 30; (299)	E + G: 127; F + P: 24; (151)^a^	E + G: 44; F + P: 12; (56)	E + G: 137; F + P: 15; (152)
Pain VAS	2.0 ± 1.2	NI	2.4 ± 1.5	2.5 ± 0.4

*AIC* Autologous iliac crest, *ALG* allograft, *ALP* alloplastic, *LB* local bone, *NA* not applied, *NI* not informed, *VAS* Visual Analogue Scale.

^a^Cited in one study.

### Meta-analysis of primary outcomes

**Figure 1:**
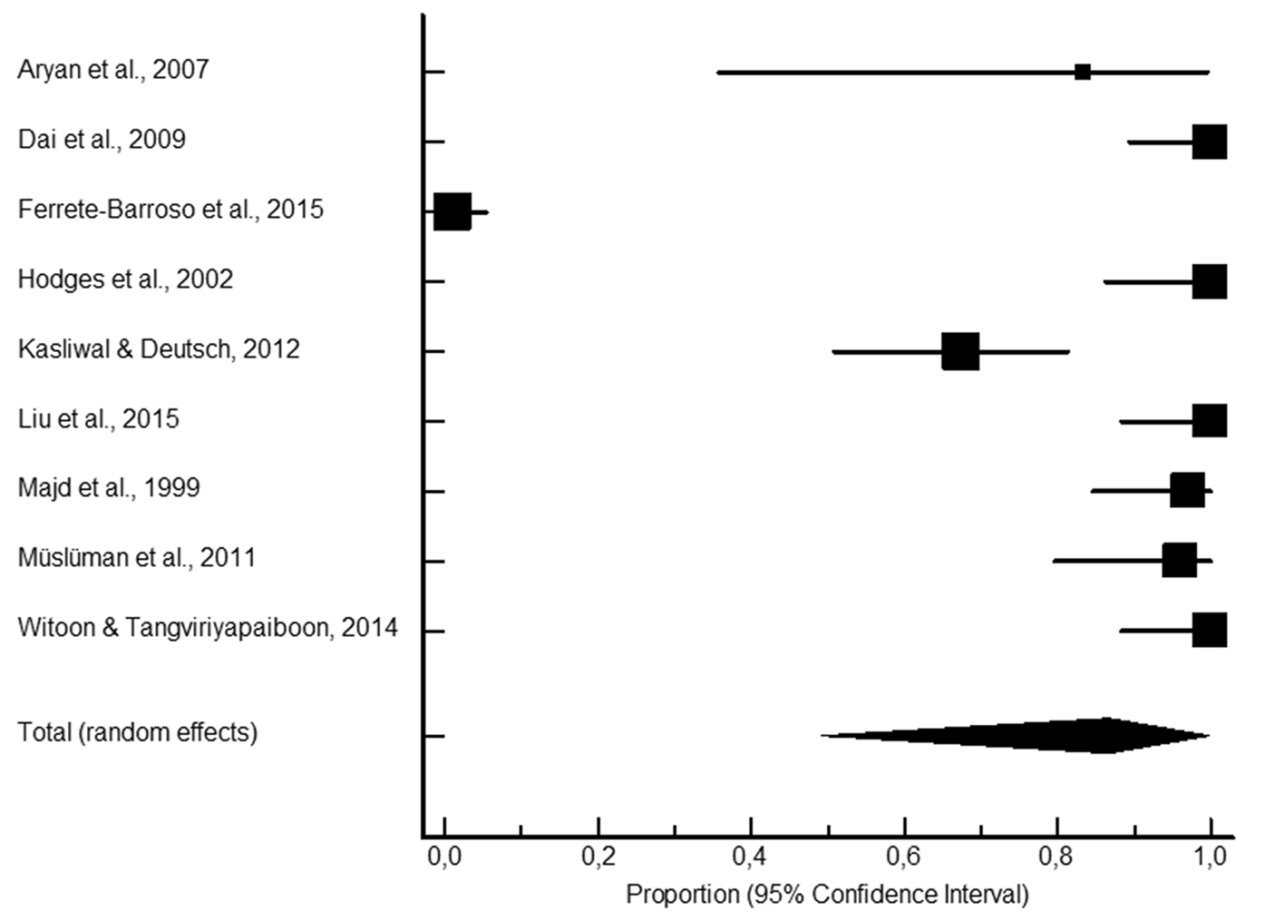
Local bone pooled proportional rate for spinal fusion.

**Figure 2:**
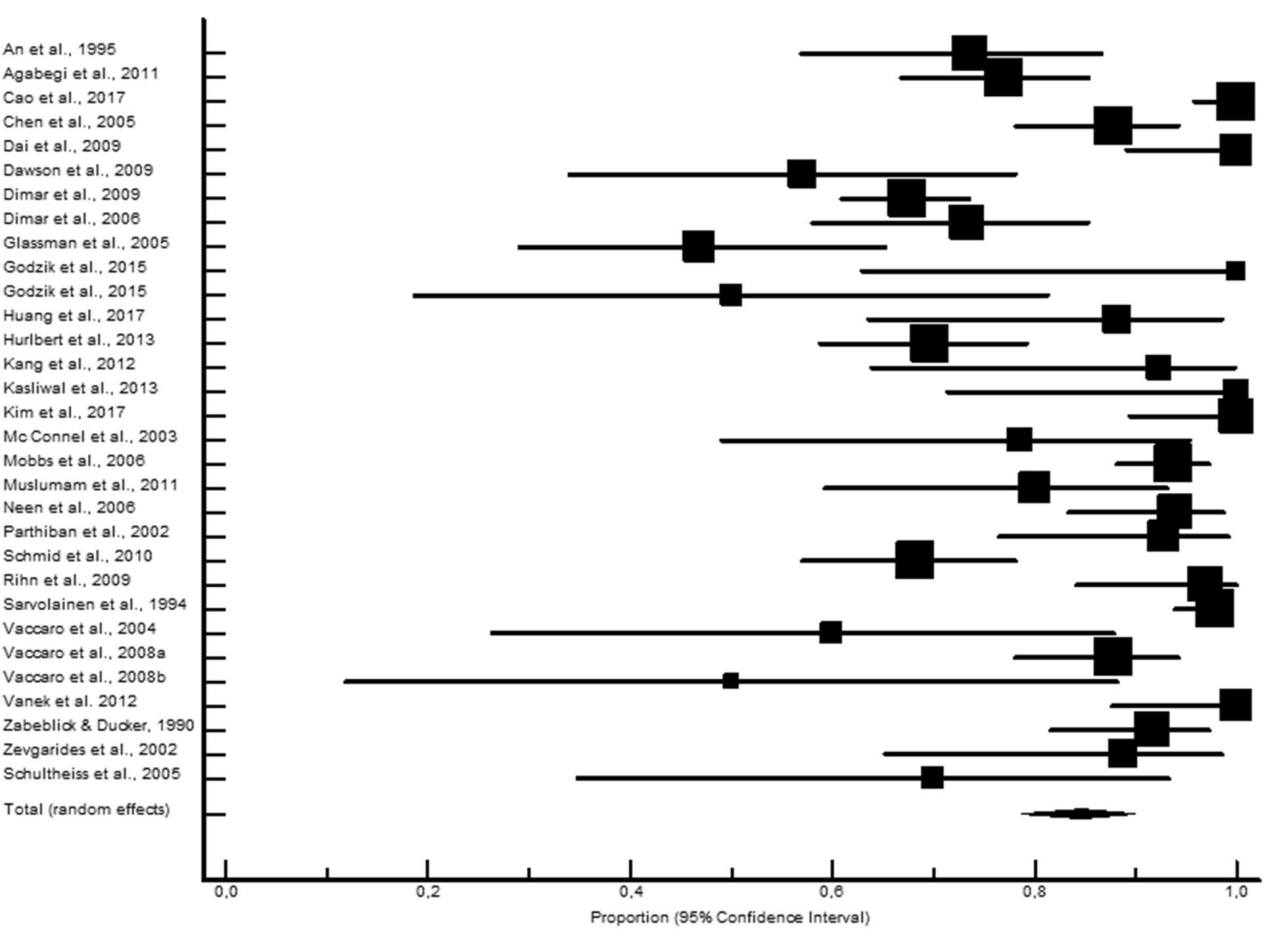
Autologous iliac crest pooled proportional rate for spinal fusion.

**Figure 3:**
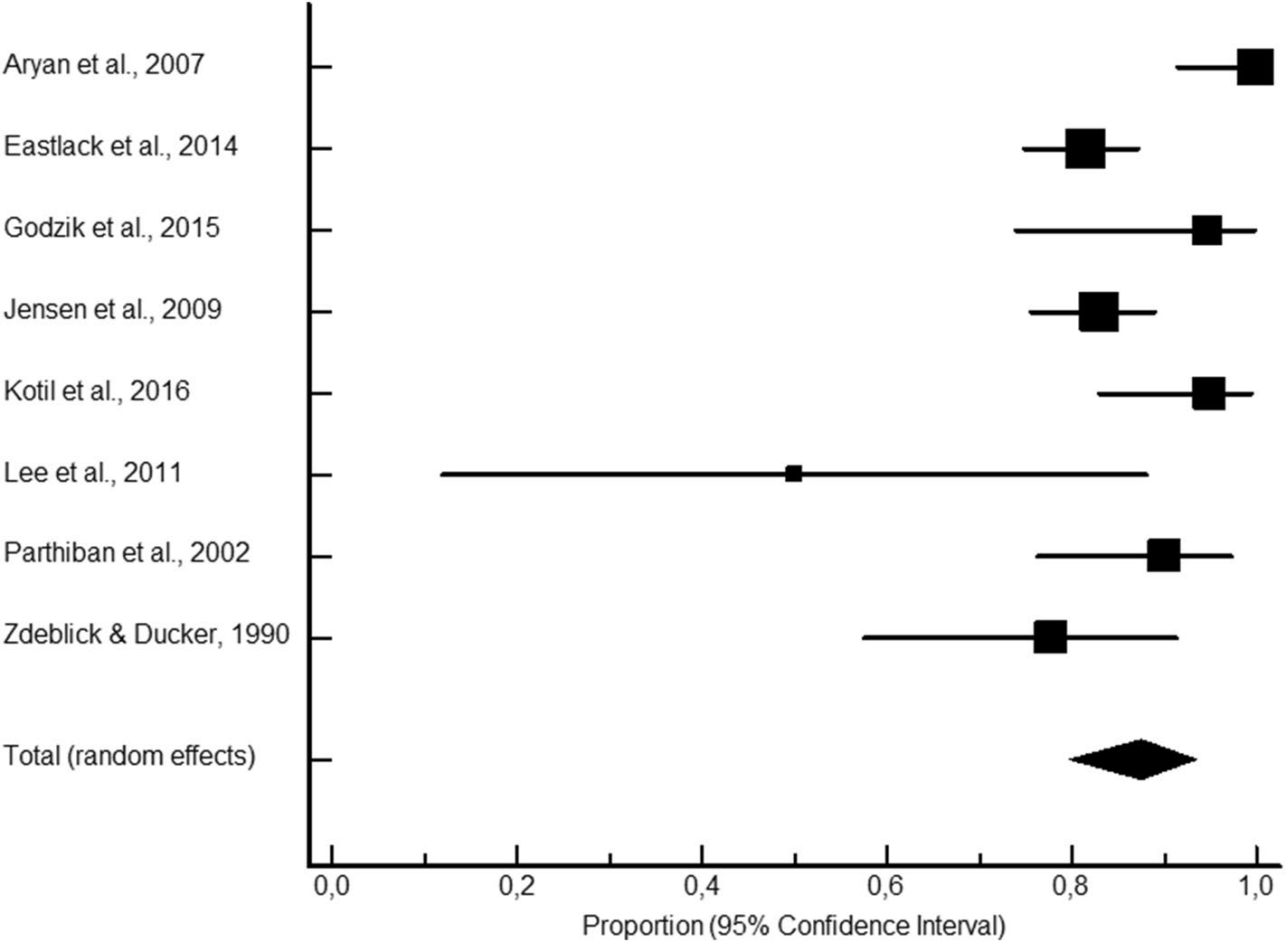
Allograft pooled proportional rate for spinal fusion.

**Figure 4:**
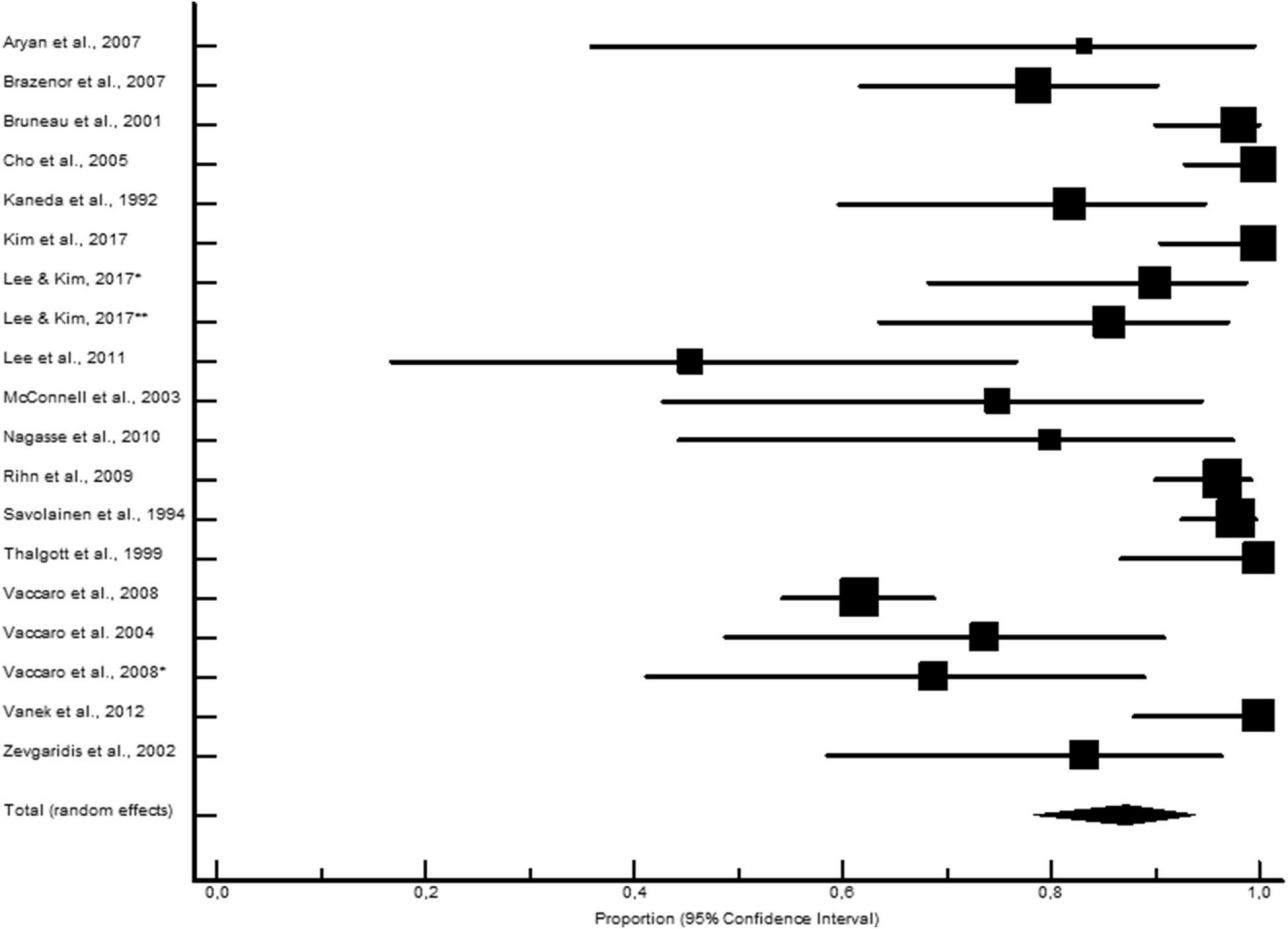
Alloplastic pooled proportional rate for spinal fusion.

LB presented significantly higher proportions of fusion rates (346 fusions out of 366; 95.3% CI 89.7–98.7; Fig. 1) compared to the AIC (2038 fusions out of 2336; 88.6% CI 84.8–91.9; Fig. 2), ALG (381 fusions out of 494; 87.8% CI 80.8–93.4: Fig. 3), and ALP (613 fusions out of 744; 85.8% CI 75.7–93.5; Fig. 4) study groups. Moderately to highly significant inconsistency (I^2^ > 50%, *P* < 0.001) was found in all proportion analyses (86.4%, 74.9%, 73.8%, and 91.6%, for AIC, LB, ALG, and ALP, respectively).

### Subgroup analysis

To determine what proportion of the summary results were driven by studies that had used grafts with metallic implants, we conducted subgroup analysis by dividing the studies into groups with grafts alone and grafts with metallic implants for all study groups. For AIC alone, the pooled proportion of fusion rates stood at 85.6% (CI 79.8–90.5, I^2^ = 87.6%), whereas AIC with metallic implants showed fusion rates of 92.3% (CI 87.6–96.0, I^2^ = 81.6%). The pooled proportion rates for LB alone and combined with metallic implants were 99.1% (CI 94.8–99.8) and 93.9% (CI 86.6–98.5, I^2^ = 78.4%), respectively. The ALG and ALP studies’ pooled proportion rates for grafts alone were 89.6% (CI 80.1–96.3, I^2^ = 70.6%) and 81.7% (CI 62.2–95.2, I^2^ = 92.6%), respectively, versus 82.1% (CI 77.6–86.2, I^2^ = 0.0%) and 91.4% (CI 76.4–99.2, I^2^ = 92.0%), respectively, for grafts combined with metallic implants.

Studies that stratified spinal fusion by follow-up period are presented in Supplementary Fig. 4. Detailed information about rates and confidence intervals are presented in Supplementary Table 4.

### Meta-analysis of secondary outcomes

Only 32 studies described rates of pseudarthrosis: 17 in the AIC group, six in the ALP group, four in the ALG group, and five in the LB group. Pseudarthrosis presented a pooled proportion of 14.2% CI 8.9–20.5%, I^2^ = 74.2%, and *P* < 0.0001 for the lumbar spine region (88 of 625 patients) versus 4.1% CI 1.6–7.7%, I^2^ = 76.6%, and *P* < 0.0001 for the cervical spine (29 of 776 patients). According to applied grafts, pseudarthrosis achieved a significantly lower pooled proportion in ALG studies (four events among 243 patients, 4.8% CI 0.1–15.7, I^2^ = 77.3%) compared to AIC studies (81 events among 851 patients, 8.6% CI 4.2–14.2, I^2^ = 84.0%), ALP studies (14 events among 153 patients, 7.1% CI 0.9–18.2, I^2^ = 78.3%), and LB studies (18 events among 154 patients, 10.3% CI 1.8–24.5, I^2^ = 81.7%).

Adverse events analysis was performed using three main categories: *pain*, *infection*, and *graft-related events* (graft collapse, fragmentation, protrusion, or breach). We also added *donor site morbidity* for the AIC sample. ALP and AIC studies described significantly more cases (80 events among 404 patients and 860 events among 2001 patients, respectively) than LB studies (20 events among 311 patients) and ALG studies (73 events among 459 patients). For our proportion analysis, we considered only events per patient. Table 4 displays our proportions analysis calculations based on the available data.

**Table 4:** Adverse events proportions analysis.

Aspects	AIC	ALG	ALP	LB
Donor site morbidity	23.2% (12.4–36.1%) I^2^ 96.3%; p < 0.0001	NA	NA	NA
Pain	23.4% (0.06–74.1%) I^2^ 99.5%; p < 0.0001	12.5% (2.2–29.7%) I^2^ 91.5%; p < 0.0001	ID	ID
Infection	5.8% (2.5–10.3%) I^2^ 84.5%; p < 0.0001	ID	10.3% (3.7–19.6%) I^2^ 74.2%; p = 0.0037	ID
Graft related	15.7% (10.4–21.9%) I^2^ 86.4%; p < 0.0001	19.8% (5.2–40.6%) I^2^ 92.5%; p < 0.0001	35.1% (14.4–59.4%) I^2^ 93.4%; p < 0.0001	7.2% (3.9–11.3%) I^2^ 29.0%; p = 0.2063

*AIC* Autologous iliac crest, *ALG* allograft, *ALP* alloplastic, *LB* local bone, *NA* not applicable, *ID* insufficient data for proportion.

## Discussion

Through our primary outcome analysis, our study showed a higher proportion of fusion rates for LB (95.3%) compared to AIC (88.6%), ALG (87.8%), and ALP (85.8%). This finding was not expected since LB has less trabecular bone, which would theoretically result in less bone marrow and less availability of the pluripotent cells and growth factors^25^. Also, LB’s limited harvestable volume narrows its surgical recommendations, and it is commonly applied to the cervical spine (which involves a smaller area to cover and less body load to sustain compared to the lumbar spine).

Our sample mainly comprised AIC (2529) patients, followed by ALP (766), ALG (516), and LB (366) patients. This size discrepancy could explain the LB fusion effect among our pooled samples, which could exacerbate LB’s effect. Moreover, most studies did not present participants baseline assessments, and since the fusion quality of distinctive grafts can diverge by age, metabolic activity, or graft-bed preparation^26,27^, confirming LB graft fusions superiority to the other studied options is challenging. Similarly, most of the reviewed studies did not follow the FDA’s guidance for spinal fusion evaluations^28^, increasing their assessment bias.

Additionally, the literature has often identified conflicting opinions regarding the optimal association between surgical techniques and patients’ underlying predictive factors for spinal fusions and spinal grafts. Other meta-analyses, that have considered assorted graft materials or surgical approaches, have demonstrated higher fusion rates using rhBMP^27,29,30^ or when grafts are associated with the anterior lumbar interbody fusion technique^31^. Moreover, minimally invasive procedures did not demonstrate fusion rate differences compared to open surgical techniques^32^.

Considering the data inconsistencies in our primary analysis, which precluded further associations (e.g., fusion rate × graft type × surgical technique), we performed a subgroup analysis of fusion rates with or without metallic implants. In this subgroup, LB presented lower fusion rates when associated with metallic implants, and this finding could be explained by LB limitations in graft volume availability^33^ and/or small patient sample.

Pseudarthrosis rates and adverse events were studied as secondary outcomes. Our pseudarthrosis analysis revealed that the reported data presented a higher proportional rate of pseudarthrosis in the lumbar spine (14.2%) than the cervical spine (4.1%), consistently with previous analyses^6^, which was explained by the increased difficulty of stabilizing areas that support higher loads^34,35^. Furthermore, our analysis of bone graft types revealed that LB presented a higher pooled proportional pseudarthrosis rate (10.5%). However, some considerations are worth mentioning. Pseudarthrosis rates were not systematically assessed across the reviewed studies (AIC 17 of 51 analyses; ALG 4 of 9 analyses; ALP 6 of 20 analyses; and LB 5 of 10 analyses), which could have exacerbated the discrepancy between patient quantity and analyzed effects. Similarly, authors’ descriptions of their results did not suggest that pseudarthrosis can be presumed to directly result from fusion rates’ missing from fusion rate analyses. Moreover, the literature did not present a conclusive role governing bone grafts’ influence on pseudarthrosis rates^6^.

Greater pseudarthrosis rates have already been associated with advanced age (because of delayed bridging maturation and increased bone resorption)^36^, degenerative disease, and construct length^6^. Longer fusions can enable loading distribution, minimizing excess motion and helping to decrease pseudarthrosis^34,37^. However, they can also increase points of load failure for each adjacent segment^34^, demand more grafts, and increase patients’ exposure to complications (due to an extensive surgical intervention). Nevertheless, our literature review examined a limited sample for this subgroup analysis, and it included many studies with moderate to high heterogeneity, reflecting pseudarthrosis evaluations’ diversity. For example, Choudhri et al.^38^ recommend CT imaging with fine-cut axial and multiplanar reconstruction to evaluate spinal fusions. Nonetheless, no radiographic gold standard is available with which to evaluate pseudarthrosis^38^ compared to open surgical exploration. Therefore, as in the literature, our review did not reveal a conclusive role governing bone grafts’ influence on pseudarthrosis rates^6^.

Moreover, many available studies presented substantial methodological flaws regarding adverse events, limiting analyses. AIC pain corresponded to a 23.4% pooled proportional rate and a significant proportion of donor site morbidity (23.2%), corroborating the previously mentioned graft drawbacks already described in the literature^11–16^. Unsurprisingly, and as we have mentioned, foreign bodies can carry some inherent risks, which could explain ALP’s higher pooled proportional rates of infection (10.2%) and graft-related events (35.1%).

Our study faced other limitations. Heterogeneity was found in different aspects of the reviewed studies’ populations. This heterogeneity arose from clinical diversity in both treatment groups, supported by insufficient analyses, a small pool of subjects, differences on assessing patients’ baseline and outcomes, and the absence of systematic reports (e.g., the use of tobacco or nonsteroidal anti-inflammatory drugs could have led to a misinterpretation of fusion rates). Moreover, a standard tool for data collection could improve data availability for fusion rate analysis and pseudarthrosis assessment. Furthermore, we did not include all available ALP grafts due to the high existent variability, which could wane proportional analysis. An example is the platelet rich plasma, which is gaining recognition as an important adjunct in the spinal graft market^39^. Finally, an overall higher RoB—which could influence appraisals of interventions effects—indicated a lack of structured randomized trials. Moreover, successful treatments should be interpreted in light of patients diminished exposure to nosocomial events, acceptable survival rates, and function after treatment.

Comparing the inputs of more than three decades of medical evolution is challenging, given technical improvements, instrumental variations, and a greater range of material. The competition for better outcomes versus materials will continue, as well the difficulty of medical updates and the discernment of industry interests. Structured clinical trials are highly encouraged to promote the availability of optimal, cost–benefit treatments for patients.

The findings of our analysis demonstrate substantial variety of spinal grafts and the need for more rigorous studies to better address and assist surgeons in choosing the best graft options. Standardized methods to evaluate spinal fusion and pseudarthrosis are encouraged.

## Supplementary Information

Supplementary Information.

## Data Availability

Upon request to the corresponding author.
