# Supplementary material for: A systematic review and meta-analysis of fusion rate enhancements and bone graft options for spine surgery
Source: Sci Rep. 2022 May 9;12:7546. doi: 10.1038/s41598-022-11551-8 (PMC9085837; doi:10.1038/s41598-022-11551-8)
Supplement: Supplementary file 1 — Supplementary Information. [file 41598_2022_11551_MOESM1_ESM.docx]

**Supplementary Figure 1.** PRISMA Meta-analysis flowchart for identifying studies focused on fusion rates in spinal fusion procedures.

Full-text articles excluded, with reasons
(n = 120)

Full-text articles assessed for eligibility
(n = 184)

Records identified through database searching
(n = 1535)

Records excluded
(n = 472)

Records screened
(n = 656)

Records after duplicates removed
(n = 656)

## Identification

## Eligibility

## Included

## Screening

Studies included in qualitative synthesis (n = 64 ^1-64^)

Studies included in quantitative synthesis (meta-analysis)
(n = 64 ^1-64^)

| **Supplementary Table 1.** Reasons of full texts exclusion. | |
| --- | --- |
| **Reason of exclusion** | **Quantity of articles** |
| Employment of 2 grafts | 33 |
| Fusion rate is not informed/not clear | 25 |
| Employment of 3 or more grafts | 20 |
| Patients under 18 years old | 13 |
| Patients with pseudarthrosis as antecedents | 9 |
| Uncertain employment of two graft types or more | 6 |
| Fusion rate by spinal level only | 5 |
| Fusion rate impaired by graft’s diversity in the same sample | 5 |
| One study of its graft type | 3* |
| Fusion rate assessed in others surgical procedures (example: rhinoplasty) | 1 |

*(Manubrium; occipital; femur head)

**Supplementary Table 2.** PRISMA checklist

| **Section and Topic** | **Item #** | **Checklist item** | **Location where item is reported** |
| --- | --- | --- | --- |
| **TITLE** | | |  |
| Title | 1 | Identify the report as a systematic review. | 01 |
| **ABSTRACT** | | |  |
| Abstract | 2 | See the PRISMA 2020 for Abstracts checklist. | 02-03 |
| **INTRODUCTION** | | |  |
| Rationale | 3 | Describe the rationale for the review in the context of existing knowledge. | 04-05 |
| Objectives | 4 | Provide an explicit statement of the objective(s) or question(s) the review addresses. | 05 |
| **METHODS** | | |  |
| Eligibility criteria | 5 | Specify the inclusion and exclusion criteria for the review and how studies were grouped for the syntheses. | 05-06 |
| Information sources | 6 | Specify all databases, registers, websites, organisations, reference lists and other sources searched or consulted to identify studies. Specify the date when each source was last searched or consulted. | 05 |
| Search strategy | 7 | Present the full search strategies for all databases, registers and websites, including any filters and limits used. | 05-06 |
| Selection process | 8 | Specify the methods used to decide whether a study met the inclusion criteria of the review, including how many reviewers screened each record and each report retrieved, whether they worked independently, and if applicable, details of automation tools used in the process. | 06 |
| Data collection process | 9 | Specify the methods used to collect data from reports, including how many reviewers collected data from each report, whether they worked independently, any processes for obtaining or confirming data from study investigators, and if applicable, details of automation tools used in the process. | 05-06 |
| Data items | 10a | List and define all outcomes for which data were sought. Specify whether all results that were compatible with each outcome domain in each study were sought (e.g. for all measures, time points, analyses), and if not, the methods used to decide which results to collect. | 06 |
|  | 10b | List and define all other variables for which data were sought (e.g. participant and intervention characteristics, funding sources). Describe any assumptions made about any missing or unclear information. | 06 |
| Study risk of bias assessment | 11 | Specify the methods used to assess risk of bias in the included studies, including details of the tool(s) used, how many reviewers assessed each study and whether they worked independently, and if applicable, details of automation tools used in the process. | 07 |
| Effect measures | 12 | Specify for each outcome the effect measure(s) (e.g. risk ratio, mean difference) used in the synthesis or presentation of results. | 07-08 |
| Synthesis methods | 13a | Describe the processes used to decide which studies were eligible for each synthesis (e.g. tabulating the study intervention characteristics and comparing against the planned groups for each synthesis (item #5)). | 07-08 |
|  | 13b | Describe any methods required to prepare the data for presentation or synthesis, such as handling of missing summary statistics, or data conversions. | 07-08 |
|  | 13c | Describe any methods used to tabulate or visually display results of individual studies and syntheses. | 07-08 |
|  | 13d | Describe any methods used to synthesize results and provide a rationale for the choice(s). If meta-analysis was performed, describe the model(s), method(s) to identify the presence and extent of statistical heterogeneity, and software package(s) used. | 07-08 |
|  | 13e | Describe any methods used to explore possible causes of heterogeneity among study results (e.g. subgroup analysis, meta-regression). | 07 |
|  | 13f | Describe any sensitivity analyses conducted to assess robustness of the synthesized results. | 07-08 |
| Reporting bias assessment | 14 | Describe any methods used to assess risk of bias due to missing results in a synthesis (arising from reporting biases). | 07 |
| Certainty assessment | 15 | Describe any methods used to assess certainty (or confidence) in the body of evidence for an outcome. | 07-08 |
| **RESULTS** | | |  |
| Study selection | 16a | Describe the results of the search and selection process, from the number of records identified in the search to the number of studies included in the review, ideally using a flow diagram. | 08 |
|  | 16b | Cite studies that might appear to meet the inclusion criteria, but which were excluded, and explain why they were excluded. | 08 |
| Study characteristics | 17 | Cite each included study and present its characteristics. | 08-09 |
| Risk of bias in studies | 18 | Present assessments of risk of bias for each included study. | 08 |
| Results of individual studies | 19 | For all outcomes, present, for each study: (a) summary statistics for each group (where appropriate) and (b) an effect estimate and its precision (e.g. confidence/credible interval), ideally using structured tables or plots. | 08-11 |
| Results of syntheses | 20a | For each synthesis, briefly summarise the characteristics and risk of bias among contributing studies. | 08 |
|  | 20b | Present results of all statistical syntheses conducted. If meta-analysis was done, present for each the summary estimate and its precision (e.g. confidence/credible interval) and measures of statistical heterogeneity. If comparing groups, describe the direction of the effect. | 08-11 |
|  | 20c | Present results of all investigations of possible causes of heterogeneity among study results. | 08-11 |
|  | 20d | Present results of all sensitivity analyses conducted to assess the robustness of the synthesized results. | 08-11 |
| Reporting biases | 21 | Present assessments of risk of bias due to missing results (arising from reporting biases) for each synthesis assessed. | NA |
| Certainty of evidence | 22 | Present assessments of certainty (or confidence) in the body of evidence for each outcome assessed. | 08-11 |
| **DISCUSSION** | | |  |
| Discussion | 23a | Provide a general interpretation of the results in the context of other evidence. | 11-14 |
|  | 23b | Discuss any limitations of the evidence included in the review. | 13-14 |
|  | 23c | Discuss any limitations of the review processes used. | 13-14 |
|  | 23d | Discuss implications of the results for practice, policy, and future research. | 15 |
| **OTHER INFORMATION** | | |  |
| Registration and protocol | 24a | Provide registration information for the review, including register name and registration number, or state that the review was not registered. | NA |
|  | 24b | Indicate where the review protocol can be accessed, or state that a protocol was not prepared. | NA |
|  | 24c | Describe and explain any amendments to information provided at registration or in the protocol. | NA |
| Support | 25 | Describe sources of financial or non-financial support for the review, and the role of the funders or sponsors in the review. | 01 |
| Competing interests | 26 | Declare any competing interests of review authors. | 01 |
| Availability of data, code and other materials | 27 | Report which of the following are publicly available and where they can be found: template data collection forms; data extracted from included studies; data used for all analyses; analytic code; any other materials used in the review. | Supplemental File |

*From:*  Page MJ, McKenzie JE, Bossuyt PM, Boutron I, Hoffmann TC, Mulrow CD, et al. The PRISMA 2020 statement: an updated guideline for reporting systematic reviews. BMJ 2021;372:n71. doi: 10.1136/bmj.n71

For more information, visit: <http://www.prisma-statement.org/>

| **Supplementary** T**able 3. MINORS quality assessment.** | | | | | | | | | | | | | |
| --- | --- | --- | --- | --- | --- | --- | --- | --- | --- | --- | --- | --- | --- |
|  | Clearly stated aim | Inclusion of consecutive patients | Prospective collection of data | Endpoints appropriate to the aim of the study | Unbiased assessment of the study endpoint | Follow-up period appropriate to the aim of the study | Loss to follow up less than 5% | Prospective calculation of the study size | **An adequate control group** | **Contemporary groups** | **Baseline equivalence of groups** | **Adequate statistical analyses** | **TOTAL** |
| **Agabegi et al., 2011^1^** | 1 | 0 | 0 | 1 | 2 | 2 | 1 | 2 | NA | NA | NA | NA | **9/16** |
| **An et al., 1995^2^** | 2 | 1 | 0 | 1 | 2 | 2 | 2 | 0 | NA | NA | NA | NA | **10/16** |
| **Aryan et al., 2007^3^** | 0 | 0 | 0 | 1 | 0 | 2 | 2 | 0 | 1 | 2 | 1 | 0 | **9/24** |
| **Brazenor, 2007^4^** | 1 | 2 | 0 | 1 | 1 | 2 | 1 | 2 | NA | NA | NA | NA | **10/16** |
| **Cao et al., 2017^7^** | 1 | 1 | 0 | 1 | 0 | 2 | 2 | 2 | NA | NA | NA | NA | **9/16** |
| **Coe, 2004^9^** | 0 | 0 | 0 | 1 | 0 | 2 | 2 | 1 | NA | NA | NA | NA | **6/16** |
| **Doria et al., 2018^14^** | 2 | 0 | 0 | 2 | 1 | 2 | 2 | 0 | NA | NA | NA | NA | **9/16** |
| **Eastlack et al., 2014^15^** | 2 | 0 | 0 | 1 | 2 | 2 | 1 | 0 | NA | NA | NA | NA | **8/16** |
| **Epstein, 2007^16^** | 1 | 2 | 0 | 1 | 2 | 2 | 1 | 0 | NA | NA | NA | NA | **9/16** |
| **Ferrete-Barroso et al., 2015^17^** | 2 | 0 | 0 | 1 | 0 | 2 | 2 | 0 | NA | NA | NA | NA | **7/16** |
| **Godzik et al., 2015^19^** | 2 | 1 | 0 | 1 | 0 | 2 | 1 | 1 | 2 | 1 | 1 | 2 | **14/24** |
| **Hodges et al., 2002^22^** | 2 | 0 | 0 | 1 | 0 | 2 | 2 | 1 | NA | NA | NA | NA | **8/16** |
| **Huang et al., 2017^23^** | 2 | 2 | 1 | 1 | 2 | 2 | 2 | 2 | NA | NA | NA | NA | **14/16** |
| **Jensen et al., 2009^25^** | 2 | 2 | 2 | 1 | 2 | 2 | 2 | 2 | NA | NA | NA | NA | **15/16** |
| **Kaneda et al., 1992^26^** | 1 | 2 | 2 | 1 | 0 | 2 | 2 | 0 | NA | NA | NA | NA | **10/16** |
| **Kasliwal et Deutsch, 2012^29^** | 2 | 0 | 0 | 1 | 0 | 2 | 1 | 1 | NA | NA | NA | NA | **7/16** |
| **Kim et al., 2017^30^** | 2 | 1 | 1 | 1 | 2 | 2 | 2 | 2 | 2 | 1 | 1 | 1 | **18/24** |
| **Kotil, 2016^31^** | 0 | 0 | 0 | 1 | 0 | 2 | 2 | 0 |  |  |  |  | **5/16** |
| **Lee et Kim, 2017^32^** | 2 | 2 | 0 | 1 | 2 | 2 | 2 | 1 | NA | NA | NA | NA | **12/16** |
| **Lee et al., 2011^33^** | 2 | 0 | 0 | 1 | 0 | 2 | 1 | 0 | 1 | 1 | 1 | 0 | **9/24** |
| **Liu et al., 2015^34^** | 2 | 0 | 0 | 1 | 1 | 2 | 2 | 2 | NA | NA | NA | NA | **10/16** |
| **Majd et al., 1999^35^** | 2 | 0 | 0 | 1 | 2 | 2 | 2 | 0 | NA | NA | NA | NA | **9/16** |
| **Mobbs et al., 2006^37^** | 2 | 1 | 0 | 1 | 0 | 2 | 2 | 0 | 2 | 1 | 1 | 0 | **12/24** |
| **Mummaneni et al. 2004^38^** | 1 | 0 | 0 | 1 | 0 | 2 | 2 | 0 | NA | NA | NA | NA | **6/16** |
| **Nagasse et al., 2010^40^** | 0 | 0 | 0 | 1 | 0 | 2 | 2 | 0 | 2 | 2 | 1 | 0 | **10/24** |
| **Neen et al., 2006^41^** | 1 | 0 | 0 | 1 | 2 | 2 | 1 | 0 |  |  |  |  | **7/16** |
| **Parthiban et al., 2002^42^** | 1 | 1 | 0 | 1 | 0 | 2 | 2 | 0 | 2 | 2 | 1 | 0 | **12/24** |
| **Rene et al., 2010^43^** | 1 | 2 | 1 | 1 | 0 | 2 | 1 | 0 | NA | NA | NA | NA |  |
| **Rihn et al., 2009^44^** | 2 | 0 | 0 | 1 | 0 | 2 | 1 | 2 | 2 | 2 | 1 | 1 | **14/24** |
| **Samartzis et al., 2004^45^** | 2 | 1 | 0 | 1 | 2 | 2 | 2 | 2 | NA | NA | NA | NA | **12/16** |
| **Savolainen et al., 1994^48^** | 2 | 1 | 0 | 1 | 2 | 2 | 1 | 0 | 2 | 1 | 1 | 0 | **13/24** |
| **Schimid et al., 2010^49^** | 1 | 2 | 1 | 1 | 0 | 2 | 2 | 1 | NA | NA | NA | NA | **10/16** |
| **Schultheiss et al., 2005^50^** | 2 | 0 | 0 | 1 | 0 | 1 | 2 | 0 | 2 | 2 | 2 | 0 | **12/24** |
| **Thalgott et al., 1999^51^** | 2 | 0 | 0 | 1 | 0 | 2 | 2 | 0 | NA | NA | NA | NA | **7/16** |
| **Vanek et al., 2012^55^** | 2 | 1 | 2 | 1 | 2 | 2 | 2 | 2 | 2 | 2 | 1 | 1 | **20/24** |
| **Weisbrod et al., 2019^56^** | 1 | 0 | 0 | 2 | 0 | 2 | 2 | 0 | NA | NA | NA | NA | **7/16** |
| **Witoon & Tangviriyapaiboon, 2014^57^** | 1 | 0 | 0 | 1 | 1 | 2 | 2 | 0 | NA | NA | NA | NA | **7/16** |
| **Yang et al., 2019^58^** | 2 | 1 | 0 | 2 | 2 | 1 | 2 | 0 | 1 | 2 | 1 | 1 | **15/24** |
| **Yang et al., 2019-2^59^** | 2 | 2 | 2 | 1 | 2 | 2 | 2 | 0 | 1 | 2 | 1 | 1 | **18/24** |
| **Yeung et al., 2019^60^** | 1 | 1 | 0 | 2 | 0 | 2 | 1 | 0 | NA | NA | NA | NA | **7/16** |
| **Yue et al., 2003^61^** | 2 | 1 | 1 | 2 | 1 | 2 | 2 | 0 | NA | NA | NA | NA | **11/16** |
| **Zdeblick & Ducker, 1991^62^** | 2 | 1 | 1 | 1 | 2 | 2 | 2 | 1 | 1 | 2 | 1 | 1 | **17/24** |
| **Zevgaridis et al., 2002^63^** | 2 | 2 | 1 | 1 | 1 | 2 | 2 | 2 | 2 | 1 | 2 | 1 | **19/24** |
| **Zhou et al., 2010^64^** | 2 | 2 | 0 | 1 | 2 | 2 | 2 | 2 | 2 | 2 | 1 | 1 | **19/24** |

NA – Not applied

**Supplementary Figure 2.** Cochrane Collaboration’s tool – Summary of risk of bias.


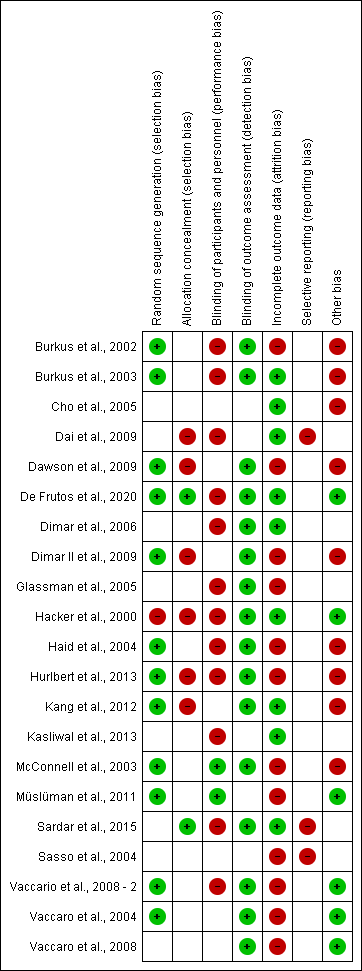


**Supplementary Figure 3.** Cochrane Collaboration’s tool – Risk of bias graphic.


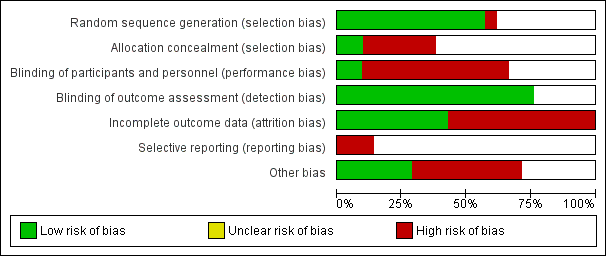


**Supplementary Figure 4.** Pooled proportion of fusion rates per graft, by follow-up.


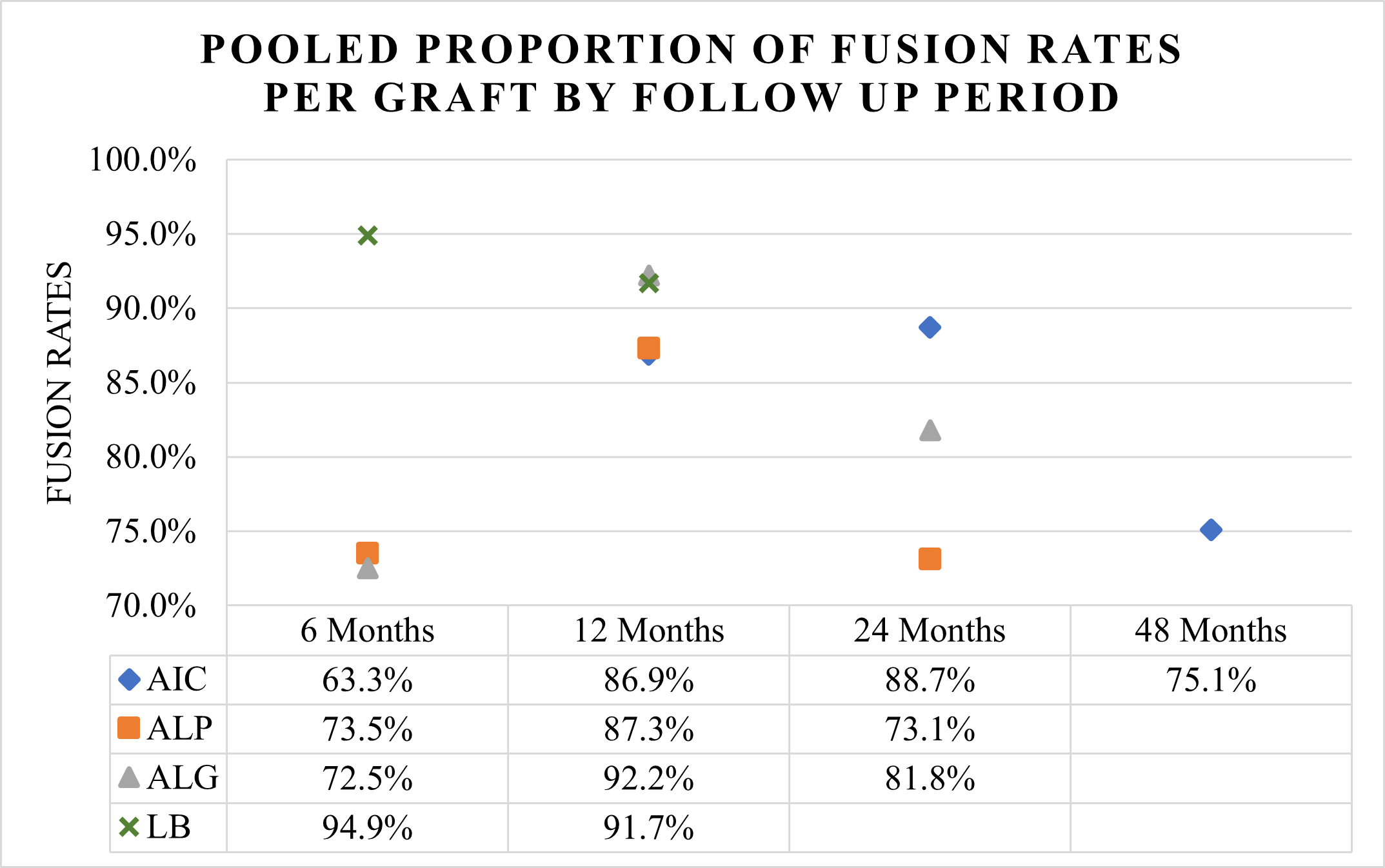


| **Supplementary Table 4. Proportion of fusion rates per graft by follow up period** | | | | |
| --- | --- | --- | --- | --- |
| **Follow up period** | Fusion rate (%), CI, I^2^, p | | | |
|  | AIC | ALG | ALP | LB |
| **6 Months** | 63.3%, 42.5 to 81.8, I^2^=96.4%, p<0,001 | 73.5%, 1.3 to 86.6, I^2^=98.1%, p<0.001 | 72.5%, 7.9 to 96.8, I^2^=97.1%, p<0.001 | 94.9%, 83.0 to 99.9, I^2^=77.2%, p=0.013 |
| **12 Months** | 86.9%, 80.7 to 91.9, I^2^=87.2%, p<0.001 | 87.3%, 80.1 to 93.1, I^2^=4.01%, p=0.373 | 92.2%, 82.7 to 98.1, I^2^=72.4%, p=0.001 | 91.7%, 75.5 to 99.6, I^2^=87.3%, p<0.001 |
| **24 Months** | 88.7%, 83.8 to 92.8, I^2^=77.8%, p<0.001 | 71.3%, 40.2 to 94.1, I^2^=67.9%, p=0.077 | 81.8%, 54.2 to 98.2, I^2^= 93.5%, p<0.001 | * |
| **48 Months** | 75.1%, 30.9 to 99.6, I^2^=79.6%, p=0.027 | NI | * | NI |

*One study only. NI: Not informed.

REFERENCES

1. Agabegi SS, Majid K, Fischgrund JS, et al. (2011) c posterolateral fusion for degenerative spondylolisthesis? Spine J. https://doi.org/10.1097/BRS.0b013e31821d289f

2. An HS, Simpson JM, Glover JM, et al. (1995) Comparison between allograft plus demineralized bone matrix versus autograft in anterior cervical fusion. A prospective multicenter study. Spine J*.* https://doi.org/10.1097/00007632-199510001-00006

3. Aryan HE, Lu DC, Acosta FLJ, et al. (2007) Bioabsorbable anterior cervical plating: initial multicenter clinical and radiographic experience. Spine J*.* https://doi.org/10.1097/01.brs.0000261489.66229.c1

4. Brazenor GA. (2007) Comparison of multisegment anterior cervical fixation using bone strut graft versus a titanium rod and buttress prosthesis: analysis of outcome with long-term follow-up and interview by independent physician. Spine J. https://doi.org/10.1097/01.brs.0000250304.24001.24

5. Burkus JK, Dorchak JD, Sanders DL. (2003) Radiographic assessment of interbody fusion using recombinant human bone morphogenetic protein type 2. Spine J*.* https://doi.org/10.1097/01.BRS.0000048469.45035.B9

6. Burkus JK, Gornet MF, Dickman CA, et al. (2002) Anterior lumbar interbody fusion using rhBMP-2 with tapered interbody cages. J Spinal Disord Tech. https://doi.org/10.1097/00024720-200210000-0000.

7. Cao L, Yang E, Xu J , et al. (2017) “Direct vision” operation of posterior atlantoaxial transpedicular screw fixation for unstable atlantoaxial fractures: A retrospective study. Medicine. https://doi.org/10.1097/MD.0000000000007054

8. Cho D-Y, Lee W-Y, Sheu P-C, et al. (2005) Cage containing a biphasic calcium phosphate ceramic (Triosite) for the treatment of cervical spondylosis. Surg Neurol. https://doi.org/110.1016/j.surneu.2004.10.016

9. Coe JD. (2004) Instrumented transforaminal lumbar interbody fusion with bioabsorbable polymer implants and iliac crest autograft. Neurosurg Focus. https://doi.org/10.3171/foc.2004.16.3.12

10. Dai LY, Jiang LS, Jiang SD. (2009) Anterior-only stabilization using plating with bone structural autograft versus titanium mesh cages for two- or three-column thoracolumbar burst fractures: a prospective randomized study. Spine J. https://doi.org/10.1097/BRS.0b013e3181a4e667

11. Dawson E, Bae HW, Burkus JK, et al. (2009) Recombinant human bone morphogenetic protein-2 on an absorbable collagen sponge with an osteoconductive bulking agent in posterolateral arthrodesis with instrumentation. A prospective randomized trial. J Bone Joint Surg Am. https://doi.org/10.2106/JBJS.G.01157

12. Dimar JR, Glassman SD, Burkus JK, et al. (2009) Clinical and radiographic analysis of an optimized rhBMP-2 formulation as an autograft replacement in posterolateral lumbar spine arthrodesis. J Bone Joint Surg Am. https://doi.org/10.2106/JBJS.H.00200

13. Dimar JR, Glassman SD, Burkus KJ, et al. (2006) Clinical outcomes and fusion success at 2 years of single-level instrumented posterolateral fusions with recombinant human bone morphogenetic protein-2/compression resistant matrix versus iliac crest bone graft. Spine J. https://doi.org/10.1097/01.brs.0000240715.78657.81

14. Doria C, Mosele GR, Balsano M, et al. (2018) Anterior decompression and plate fixation in treatment of cervical myelopathy: A multicentric retrospective review. Acta Orthop Traumatol Turc. https://doi.org/10.1016/j.aott.2017.12.003

15. Eastlack RK, Garfin SR, Brown CR, et al. (2014) Osteocel Plus cellular allograft in anterior cervical discectomy and fusion: evaluation of clinical and radiographic outcomes from a prospective multicenter study. Spine J. https://doi.org/10.1097/BRS.0000000000000557

16. Epstein NE. (2007) Complication avoidance in 116 dynamic-plated single-level anterior corpectomy and fusion. J Spinal Disord Tech. https://doi.org/10.1097/01.bsd.0000248257.10284.3b

17. Ferrete-Barroso AM, González-Díaz R, Losada-Viñas JI. (2015) Anterior cervical arthrodesis using a vertebral body autograft. Rev Esp Cir Ortop Traumatol. https://doi.org/10.1016/j.recot.2014.09.003

18. Glassman SD, Dimar JR, Carreon LY, et al. (2005) Initial fusion rates with recombinant human bone morphogenetic protein-2/compression resistant matrix and a hydroxyapatite and tricalcium phosphate/collagen carrier in posterolateral spinal fusion. Spine J. https://doi.org/10.1097/01.brs.0000172157.39513.80

19. Godzik J, Ravindra VM, Ray WZ, et al. (2015) Comparison of structural allograft and traditional autograft technique in occipitocervical fusion: radiological and clinical outcomes from a single institution. J Neurosurg Spine. https://doi.org/10.3171/2014.12.SPINE14535

20. Hacker RJ. (2000) A randomized prospective study of an anterior cervical interbody fusion device with a minimum of 2 years of follow-up results. J Neurosurg. https://doi.org/10.3171/spi.2000.93.2.0222

21. Haid RW, Branch CL, Alexander JT, et al. (2004) Posterior lumbar interbody fusion using recombinant human bone morphogenetic protein type 2 with cylindrical interbody cages. Spine J. https://doi.org/10.1016/j.spinee.2004.03.025

22. Hodges SD, Humphreys SC, Eck JC, et al. (2002) A modified technique for anterior multilevel cervical fusion. J Orthop Sci. https://doi.org/10.1007/s007760200053

23. Huang D-G, Zhang X-L, Hao D-J et al. (2017) Posterior atlantoaxial fusion with a screw-rod system: Allograft versus iliac crest autograft. Clin Neurol Neurosurg. https://doi.org/10.1016/j.clineuro.2017.10.002

24. Hurlbert RJ, Alexander D, Bailey S, et al. (2013) rhBMP-2 for posterolateral instrumented lumbar fusion: a multicenter prospective randomized controlled trial. Spine J. https://doi.org/10.1097/BRS.0000000000000007

25. Jensen WK, Moore TA, Tribus CB, et al. (2009) Use of patella allograft for anterior cervical diskectomy and fusion. J Spinal Disord Tech. https://doi.org/10.1097/BSD.0b013e3181844d8e

26. Kaneda K, Asano S, Hashimoto T et al. (1992) The treatment of osteoporotic-posttraumatic vertebral collapse using the Kaneda device and a bioactive ceramic vertebral prosthesis. Spine J*.* https://doi.org/10.1097/00007632-199208001-00015

27. Kang J, An H, Hilibrand A, et al. (2012) Grafton and local bone have comparable outcomes to iliac crest bone in instrumented single-level lumbar fusions. Spine J. https://doi.org/10.1097/BRS.0b013e31823ed817

28. Kasliwal MK, Baskin DS, Traynelis VC. (2013) Failure of porous tantalum cervical interbody fusion devices: two-year results from a prospective, randomized, multicenter clinical study. J Spinal Disord Tech. https://doi.org/10.1097/BSD.0b013e318241e70f

29. Kasliwal MK, Deutsch H. (2012) Clinical and radiographic outcomes using local bone shavings as autograft in minimally invasive transforaminal lumbar interbody fusion. World Neurosurg. https://doi.org/10.1016/j.wneu.2011.05.049

30. Kim S-H, Lee J-K, Jang J-W, et al. (2017) Polyetheretherketone cage with demineralized bone matrix can replace iliac crest autografts for anterior cervical discectomy and fusion in subaxial cervical spine injuries. J Korean Neurosurg Soc. https://doi.org/10.3340/jkns.2015.0203.014

31. Kotil K. (2016) Replacement of vertebral lamina (laminoplasty) in surgery for lumbar isthmic spondylolisthesis: 5-year follow-up results. Asian Spine J. https://doi.org/10.4184/asj.2016.10.3.443

32. Lee DD, Kim JY. (2017) A comparison of radiographic and clinical outcomes of anterior lumbar interbody fusion performed with either a cellular bone allograft containing multipotent adult progenitor cells or recombinant human bone morphogenetic protein-2. J Orthop Surg Res. https://doi.org/10.1186/s13018-017-0618-8

33. Lee Y-P, Ghofrani H, Regev GJ, et al. (2011) A retrospective review of long anterior fusions to the sacrum. Spine J. https://doi.org/10.1016/j.spinee.2011.02.004

34. Liu J, Xiong X, Long X, et al. (2015) A new source of structural autograft for ACDF surgery: cervical laminae. Int J Clin Exp Med. https://doi.org/10.1055/s-0035-1554143

35. Majd ME, Vadhva M, Holt RT. (1999) Anterior cervical reconstruction using titanium cages with anterior plating. Spine J. https://doi.org/10.1097/00007632-199908010-00016

36. McConnell JR, Freeman BJ, Debnath UK, et al. (2003) A prospective randomized comparison of coralline hydroxyapatite with autograft in cervical interbody fusion. Spine J*.* https://doi.org/10.1097/01.BRS.0000048503.51956.E1

37. Mobbs RJ, Rao P, Chandran NK. (2007) Anterior cervical discectomy and fusion: analysis of surgical outcome with and without plating. J Clin Neurosci. https://doi.org/10.1016/j.jocn.2006.04.003

38. Mummaneni P V., Pan J, Haid RW, et al. (2004) Contribution of recombinant human bone morphogenetic protein—2 to the rapid creation of interbody fusion when used in transforaminal lumbar interbody fusion: a preliminary report. J Neurosurg Spine. https://doi.org/10.3171/spi.2004.1.1.0019

39. Müslüman AM, Yılmaz A, Cansever T, et al. (2011) Posterior lumbar interbody fusion versus posterolateral fusion with instrumentation in the treatment of low-grade isthmic spondylolisthesis: midterm clinical outcomes. J Neurosurg Spine. https://doi.org/10.3171/2010.11.SPINE10281

40. Nagasse Y, Yamazato C, Oliveira FM, et al. (2010) Retrospective study of cervical arthrodesis with autograft versus hydroxyapatite graft. Coluna. https://doi.org/10.1590/S1808-18512010000400017

41. Neen D, Noyes D, Shaw M, et al. (2006) Healos and bone marrow aspirate used for lumbar spine fusion: a case controlled study comparing healos with autograft. Spine J. https://doi.org/10.1097/01.brs.0000232028.97590.12

42. Parthiban JKBC, Singhania BK, Ramani PS. (2002) A radiological evaluation of allografts (ethylene oxide sterilized cadaver bone) and autografts in anterior cervical fusion. Neurol India 50:17–22.

43. Rene S, Dietmar K, Peter S, et al. (2010) PLIF in thoracolumbar trauma: technique and radiological results. Eur Spine J. https://doi.org/10.1007/s00586-010-1362-5

44. Rihn JA, Patel R, Makda J, et al. (2009) Complications associated with single-level transforaminal lumbar interbody fusion. Spine J. https://doi.org/10.1016/j.spinee.2009.04.004

45. Samartzis D, Shen FH, Lyon C, et al. (2004) Does rigid instrumentation increase the fusion rate in one-level anterior cervical discectomy and fusion? Spine J. https://doi.org/10.1016/j.spinee.2004.04.010

46. Sardar Z, Alexander D, Oxner W, et al. (2015) Twelve-month results of a multicenter, blinded, pilot study of a novel peptide (B2A) in promoting lumbar spine fusion. J Neurosurg Spine. https://doi.org/10.3171/2013.11.SPINE121106

47. Sasso RC, Kitchel SH, Dawson EG. (2004) A prospective, randomized controlled clinical trial of anterior lumbar interbody fusion using a titanium cylindrical threaded fusion device. Spine J. https://doi.org/10.1097/01.BRS.0000107007.31714.77

48. Savolainen S, Usenius JP, Hernesniemi J. (1994) Iliac crest versus artificial bone grafts in 250 cervical fusions. Acta Neurochir. https://doi.org/10.1007/BF01400873

49. Schmid R, Krappinger D, Seykora P, et al. (2010) PLIF in thoracolumbar trauma: technique and radiological results. Eur spine J. https://doi.org/10.1007/s00586-010-1362-5

50. Schultheiss M, Sarkar M, Arand M, et al. (2005) Solvent-preserved, bovine cancellous bone blocks used for reconstruction of thoracolumbar fractures in minimally invasive spinal surgery-first clinical results. Eur Spine J. https://doi.org/10.1007/s00586-004-0764-7

51. Thalgott JS, Fritts K, Giuffre JM, et al. (1999) Anterior interbody fusion of the cervical spine with coralline hydroxyapatite. Spine J. https://doi.org/10.1097/00007632-199907010-00005

52. Vaccaro AR, Patel T, Fischgrund J, et al. (2004) A pilot study evaluating the safety and efficacy of OP-1 Putty (rhBMP-7) as a replacement for iliac crest autograft in posterolateral lumbar arthrodesis for degenerative spondylolisthesis. Spine J. https://doi.org/10.1097/01.brs.0000137062.79201.98

53. Vaccaro AR, Whang PG, Patel T, et al. (2008) The safety and efficacy of OP-1 (rhBMP-7) as a replacement for iliac crest autograft for posterolateral lumbar arthrodesis: minimum 4-year follow-up of a pilot study. Spine J. https://doi.org/10.1016/j.spinee.2007.03.012

54. Vaccaro AR, Lawrence JP, Patel T, et al. (2008) The safety and efficacy of OP-1 (rhBMP-7) as a replacement for iliac crest autograft in posterolateral lumbar arthrodesis: a long-term (4 Years) pivotal study. Spine J. https://doi.org/10.1097/BRS.0b013e31818a314d

55. Vanek P, Bradac O, DeLacy P, et al. (2012) Comparison of 3 fusion techniques in the treatment of the degenerative cervical spine disease. Is stand-alone autograft really the “gold standard?”: prospective study with 2-year follow-up. Spine J. https://doi.org/10.1097/BRS.0b013e31825413fe

56. Weisbrod LJ, Arnold PM, Leever JD. (2019) Radiographic and CT evaluation of recombinant human bone morphogenetic protein-2-assisted cervical spinal interbody fusion. Clin Spine Surg. https://doi.org/10.1097/BSD.0000000000000720

57. Witoon N, Tangviriyapaiboon T. (2014) Clinical and radiological outcomes of segmental spinal fusion in transforaminal lumbar interbody fusion with spinous process tricortical autograft. Asian Spine J*.* https://doi.org/10.4184/asj.2014.8.2.170

58. Yang J-S, Chen H, Chu L, et al. (2019) Clinical and radiological results comparison of allograft and polyetheretherketone cage for one to two-level anterior cervical discectomy and fusion: A CONSORT-compliant article. World Neurosurg. https://doi.org/10.1097/MD.0000000000017935

59. Yang J-S, Chen H, Chu L, et al. (2019) Does Additional bone grafting of atlantoaxial joint Increase bone fusion rate of iliac crest autograft in posterior occipitocervical fusion? Retrospective, controlled study with 2-year follow-up. World Neurosurg*.* https://doi.org/10.1016/j.wneu.2018.12.153

60. Yeung KKL, Cheung PWH, Cheung JPY. (2019) Anterior cervical discectomy and fusion for cervical myelopathy using stand-alone tricortical iliac crest autograft: Predictive factors for neurological and fusion outcomes. J Orthop Surg. https://doi.org/10.1177/2309499019869166

61. Yeu WM, Tay BK, Kasinathan ST. (2003) Patellar allografts in anterior cervical fusion - a two-year clinical and radiographic study. Singapore Med J. 2003;44:521–525.

62. Zdeblick TA, Ducker TB. (1991) The use of freeze-dried allograft bone for anterior cervical fusions. Spine J 16:726–729.

63. Zevgaridis D, Thome C, Krauss JK. (2002)Prospective controlled study of rectangular titanium cage fusion compared with iliac crest autograft fusion in anterior cervical discectomy. Neurosurg Focus. https://doi.org/10.3171/foc.2002.12.1.3

64. Zhou J, Xia Q, Dong J, et al. (2011) Comparison of stand-alone polyetheretherketone cages and iliac crest autografts for the treatment of cervical degenerative disc diseases. Acta Neurochir. https://doi.org/10.1007/s00701-010-0821-4
